# Comparison of heat acclimation after once daily and thrice daily heat exposures in healthy adults

**DOI:** 10.14814/phy2.70796

**Published:** 2026-02-26

**Authors:** Alejandro M. Rosales, Jessica L. Moler, Andrew C. Engellant, Alice L. Held, Brent C. Ruby, Dustin R. Slivka

**Affiliations:** ^1^ School of Integrative Physiology and Athletic Training, Montana Center for Work Physiology and Exercise Metabolism University of Montana Missoula Montana USA

**Keywords:** acclimatization, estradiol, progesterone, skin blood flow, skin conductivity, thermoregulation

## Abstract

Determine how matched duration but varied exposure scheduling impacts heat acclimation in male and female adults. Thirty males and thirty females walked daily (7 days, 38°C, 60% RH, 6.1 METs) in one of four groups (sustained males/females [SM/SF], periodic males/females [PM/PF]). SM/SF performed 90‐min exposures; PM/PF completed three 30‐min exposures 3 h apart. Females had similar ovarian‐hormone fluctuation. Acclimation markers were assessed within the first 30‐min exposure on days 1, 4, and 7. SM/SF rectal temperature decreased from day 1 to days 4 and 7 (37.5 ± 0.3°C, 37.3 ± 0.3°C, 37.2 ± 0.3°C, *p* < 0.001) and further decreased from day 4 to 7 (*p* = 0.011). PM/PF rectal temperature was unchanged between days 1, 4, and 7 (37.4 ± 0.3°C, 37.4 ± 0.3°C, 37.3 ± 0.3°C, *p* > 0.05). SM/SF 3‐site surface temperature decreased from day 1 to days 4 and 7 (37.1 ± 0.5°C, 36.9 ± 0.4°C, 36.8 ± 0.4°C, *p* < 0.001) but was unchanged from day 4 to 7 (*p* = 0.090). PM/PF 3‐site surface temperature was unchanged from day 1 (37.0 ± 0.4°C) to days 4 (37.0 ± 0.4°C, *p* = 0.726) and 7 (36.9 ± 0.4°C, *p* = 0.109) but decreased from day 4 to 7 (*p* = 0.013). Females had higher rectal (*p* < 0.001) and 3‐site surface (*p* = 0.036) temperatures than males throughout acclimation. Thrice‐daily exposures are not as effective at inducing heat adaptations compared to once‐daily exposures. Sex differences persisted throughout acclimation without altering adaptations.

## 1 INTRODUCTION

Contemporary safety recommendations in the heat advise using systematic heat exposures and exercise to become acclimated/acclimatized (Sawka et al., 2003; Centers for Disease Control and Prevention (CDC), 2023; Occupational Health and Safety Administration (OSHA), 2025; Eifling et al., 2024; Périard et al., 2015). Common physiological changes during a heat acclimation regimen include: plasma volume expansion (Bass et al., 1955; Willmott et al., 2016), increased sweat rate (Allan & Wilson, 1971; Cohen & Gisolfi, 1982; Fox et al., 1964; Frye & Kamon, 1981; Hellon et al., 1956; Wyndham et al., 1965), earlier onset of sweat (Nadel et al., 1974; Périard et al., 2015), and more dilute sweat composition (Allan & Wilson, 1971; Buono et al., 2007, 2018) that lead to lower steady state maintenance of core temperature (Barnett & Maughan, 1993; Bean et al., 1943; Cohen & Gisolfi, 1982; Duncan, 1966; Frye & Kamon, 1981; Nadel et al., 1974; Robinson et al., 1943; Wyndham et al., 1965), skin temperature (Barnett & Maughan, 1993; Cohen & Gisolfi, 1982; Duncan, 1966; Frye & Kamon, 1981; Robinson et al., 1943), and heart rate (Barnett & Maughan, 1993; Bean et al., 1943; Cohen & Gisolfi, 1982; Duncan, 1966; Frye & Kamon, 1981; Robinson et al., 1943; Wyndham et al., 1965). Each physiological change is considered an essential marker of heat acclimation. Absence of one or more of these markers suggests incomplete preparedness for physical exertion in hot environments. Although heat acclimation markers are well established, there are concerns with current heat acclimation recommendations that require further assessment.

The most critical considerations for heat acclimation are the magnitude of incurred heat stress due to a combination of the ambient temperature, exposure session schedule, and exercise. Adaptations from serial heat stresses generally occur when a critical core temperature threshold (>38.5°C) is breached (Amorim et al., 2008; Gibson et al., 2014) and sustained (Lind & Bass, 1963) alongside rises in skin temperature that prompt heat dissipation mechanisms. Thus, current recommendations encourage, at minimum, a consecutive 7‐day heat acclimation protocol (Sawka et al., 2003; Centers for Disease Control and Prevention (CDC), 2023; Occupational Health and Safety Administration (OSHA), 2025; Eifling et al., 2024; Périard et al., 2015) with 1–2 h of sustained aerobic exercise (Sawka et al., 2003; Occupational Health and Safety Administration (OSHA), 2025; Eifling et al., 2024; Périard et al., 2015). However, some recommendations suggest that heat exposures can be partitioned into smaller periodic sessions (Sawka et al., 2003; Centers for Disease Control and Prevention (CDC), 2023; Occupational Health and Safety Administration (OSHA), 2025). The assumption being that meeting total daily duration minimums (1–2 h) but with shorter periodic heat exposure sessions permits adaptations equivalent to otherwise identical continuous sessions. The rationale for separating heat exposures into smaller sessions is to minimize fatigue from the dual stressors of exercise and heat exposure (Périard et al., 2015) as well as abate early risks of heat‐related illness when beginning acclimation. The permissible interval between heat exposure sessions that still improves heat acclimation markers is not expressly established. Overlong separation intervals (days) limit heat adaptations (Barnett & Maughan, 1993) and or prolongs the time course (Fein et al., 1975; Gill & Sleivert, 2001; Lind & Bass, 1963). Multiple shorter exposures within a day may elicit equivalent adaptations to conventional protocols or at least induce select physiological adaptations to prepare for more comprehensive acclimation strategies.

An additional concern with current heat acclimation guidelines is female underrepresentation in thermoregulatory research (Hutchins et al., 2021) as current recommendations are largely derived from data collected in males (Wickham et al., 2021). Female physiology purportedly modifies heat dissipation and causes greater susceptibility for exertional heat related illnesses compared to males (Alele et al., 2020; Kazman et al., 2015; van Steen et al., 2019). Yet, many of the abovementioned standards of heat acclimation consistently translate to female physiology (Cohen & Gisolfi, 1982; Fein et al., 1975; Frye & Kamon, 1981; Giersch et al., 2025; Sawka et al., 1983; Shapiro et al., 1980; Weinman et al., 1967; Wyndham et al., 1965) except for an increased sweat rate (Giersch et al., 2025; McGlynn et al., 2022; Sawka et al., 1983; Shapiro et al., 1980; Weinman et al., 1967) or a decrease in core temperature (McGlynn et al., 2022; Shapiro et al., 1980). Some hold that females equivalently manage heat stress to males by relying on sex‐specific thermoregulatory action (Giersch et al., 2025; McGlynn et al., 2022; Weinman et al., 1967; Wyndham et al., 1965) via skin blood flow (SkBF) increases. How heat acclimation is evaluated between sexes may require attention to sex‐specific heat dissipation mechanisms that ultimately result in parallel heat stress management. Variations in heat dissipation due to female physiology may be less consequential during or following heat acclimation.

The purpose of this investigation is a simultaneous two‐part question of heat acclimation progression based on well‐established markers of thermoregulation. The first portion will specifically examine how two duration and absolute workload matched, 7‐day heat exposure regimens influence markers of heat acclimation: sustained (7 visits, 1 × 90‐min daily heat exposure session) and periodic (21 visits, 3 × 30‐min daily heat exposure sessions). The second portion will examine sex differences (males vs. females) during heat acclimation under the broader duration and absolute workload matched experimental design. We hypothesize that the sustained exposure schedule will induce optimal heat adaptations compared to the periodic exposure regardless of sex.

## 2 MATERIALS AND METHODS

### 2.1 Participants

Male (*n* = 30) and female (*n* = 30) participants (Table 1) were recruited from the surrounding University area to complete a consecutive 7‐day heat exposure protocol. All methodology was approved by the University of Montana Institutional Review Board and the Air Force Human Research Protection Office. Methodology was also completed in accord with the Declaration of Helsinki. Participants were informed of the procedures and risks associated with participation prior to providing written informed consent. Participants were considered apparently healthy based on the American College of Sports Medicine's pre‐participation questionnaire and reported completing at least 30 min of exercise, 3 days a week, for the preceding 3 months. All testing was completed within a 2‐year period during the cooler ambient temperature northern hemisphere months (46°51′43.8″N 113°59′01.0″W, October–April).

**TABLE 1:** Descriptive data for each heat acclimation group.

	Sustained male (SM)	Sustained female (SF)	Periodic male (PM)	Periodic female (PF)
Age (years)	28 ± 7	27 ± 5	27 ± 6	24 ± 4
Weight (kg)	82.5 ± 13.5	62.9 ± 9.6*	81.5 ± 13.8	63.3 ± 8.9*
Height (cm)	179.9 ± 6.8	167.7 ± 5.7*	177.2 ± 9.9	167.0 ± 5.7*
Body surface area (m^2^)	2.02 ± 0.17	1.71 ± 0.15*	2.00 ± 0.18	1.71 ± 0.14*
Sum of 7 (mm)	99.1 ± 52.3	111.5 ± 38.3	101.3 ± 47.4	108.5 ± 34.2
Body fat (%)	13.8 ± 7.5	22.0 ± 5.7*	14.1 ± 6.5	21.4 ± 5.2*
$\dot{V}O_{2}$ peak (mL·kg^−1^·min^−1^)	50.5 ± 11.1	39.9 ± 5.2*	49.2 ± 8.3	40.6 ± 3.9*
$\dot{V}O_{2}$ peak (mL·kg FFM^−1^·min^−1^)	57.8 ± 11.3	51.2 ± 5.9*	57.1 ± 7.2	51.7 ± 3.9*
$\dot{V}O_{2}$ peak (L·min^−1^)	4.05 ± 0.75	2.50 ± 0.44*	3.93 ± 0.43	2.57 ± 0.42*
Heat exposure work (J)	227,870 ± 37,182	173,911 ± 26,410*	225,290 ± 38,050	175,003 ± 24,733*

*Note*: Data presented as mean ± SD.

Abbreviation: FFM, fat‐free mass.

* *p* < 0.05 from males.

### 2.2 Preliminary testing

Preliminary testing was conducted following an overnight fast and while abstaining from alcohol and caffeine during the preceding 24 h. Preliminary testing consisted of a nude body weight, height, 7‐site skinfold measurement, and peak oxygen uptake assessment. Harpenden calipers (Baty International, Bradford, West Yorkshire, England) were used to measure skinfolds in duplicate at the chest, midaxillary, triceps, subscapular, abdomen, and suprailiac to quantify body fat percentage from sex‐specific equations (Jackson & Pollock, 1985; Ligouri et al., 2022). Height and weight were used to estimate body surface area (Mosteller, 1987). Peak oxygen uptake was assessed with a graded exercise test to volitional exhaustion on a motorized treadmill (F3, Life Fitness, Rosemont, IL, USA) with expired gas measurements on a calibrated metabolic cart (TrueOne 2400, ParvoMedics, Salt Lake City, UT, USA). Heart rate and Borg rating of perceived exertion (Borg, 1982) were recorded throughout the graded exercise test.

### 2.3 Experimental overview

**FIGURE 1:**
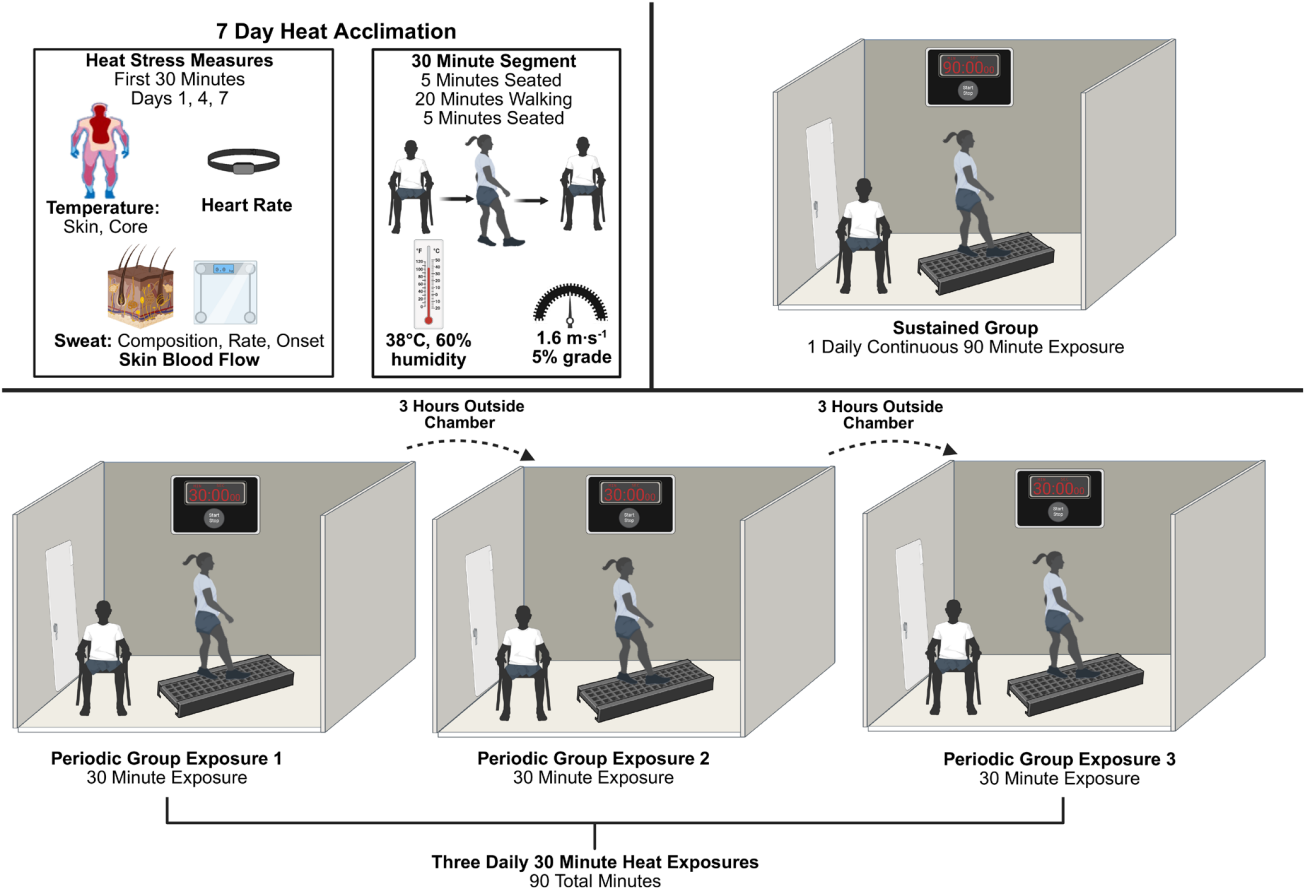
7‐day heat acclimation schematic for the sustained and periodic groups.

Experimental testing (Figure 1) occurred over 7 consecutive days with 90 min of daily heat exposure in an environmental chamber (Tescor Incorporated, Warminster, Pennsylvania, USA) set to a nearly uncompensable heat stress (Eichna et al., 1945; National Weather Service (NWS), 2025) of 38°C with 60% relative humidity (38.4 ± 0.9°C, 66.7 ± 3.2% RH). Once beginning the heat exposures, participants were instructed to abstain from alcohol and exercise outside of the protocol until study conclusion.

The experimental objective was to match overall heat exposure duration, ambient temperature, relative humidity, and exercise workload to compare the acclimation progression of two contrarian exposure schedules. Participants were divided into two heat exposure groups (sustained, periodic) based on their scheduling availability before being sub‐divided by sex. Efforts were made to ensure consistent session start times throughout the week as well as an equal representation of each group being studied at any time. The four groups were sustained male (SM), sustained female (SF), periodic male (PM), and periodic female (PF). The sustained groups completed 7 consecutive days of uninterrupted 90‐min heat exposures. The periodic groups also completed 7 consecutive days of 90‐min heat exposures but instead experienced it as three daily 30‐min sessions separated by at least 3 h so body temperature could return towards resting values. The periodic exposures were, by design, shorter sessions temporally equivalent to the longer sustained exposures.

For between group consistency, each 30‐min heat exposure segment was identical, comprised of 5 min seated rest, 20 min walking at 1.6 m·s^−1^ with 5% grade (4Front, Woodway, Waukesha, WI, USA), and 5 min seated rest. A task oriented absolute workload of 6.1 METs was chosen as a tolerable exercise intensity to complement the ambient heat load without primarily limiting session completion. All participants wore a t‐shirt, shorts, and shoes.

### 2.4 Testing: Days 1, 4, and 7

Thermoregulatory markers were assessed on days 1, 4, and 7 during the first 30 min session of the day for the periodic group and the first 30 min of the 90‐min exposure session in the sustained group. Test days began with ingestion of a gastrointestinal temperature telemetry pill at least 4 h prior to heat exposure (e‐Celsius Performance, BodyCAP, Hérouville Saint‐Clair, France; ±0.1°C accuracy). Gastrointestinal temperature was measured every 5 min. In recognition that the primary 30‐min measurement period would exclude peak heat stress, telemetry pills extended heat stress examination to include the entire 90‐min of heat exposure. Telemetry pills captured peak gastrointestinal temperature, which was not necessarily the highest value during heat exposure, but rather the highest value during the exposure or immediately upon heat chamber exit. Due to early capsule excretion or other technical issues, 162 days of usable data for peak gastrointestinal temperature (*n* = 54) were available.

Upon laboratory arrival, participants sat for 5 min while an 8–10 mL blood sample was collected from an antecubital vein (days 1 and 7 only). A nude weight was then collected prior to rectal thermistor (RET‐1, Physitemp, Clifton, NJ, USA; ±0.1°C accuracy) self‐insertion 12–15 cm past the anal sphincter. Participants were instrumented with three skin thermistors (SST‐1, Physitemp, Clifton, NJ, USA; ±0.1°C accuracy). Skin thermistors were adhered using double sided stick discs on the chest (8–10 cm from midline between sternum and xiphoid process on left pectoralis major), forearm (midline of left posterior forearm on extensor digitorum), and lower leg (left tibialis anterior in line with lower gastrocnemius head) for skin temperature measures. Participants also donned a chest strap heart rate monitor (T34, Polar Electro Inc., Bethpage, NY, USA). Participants were further instrumented with a laser doppler flowmetry probe (MSP100XP, ADInstruments, Colorado Springs, CO, USA) immediately distal to the abovementioned forearm skin thermistor using double sided stick discs for skin blood flow (SkBF) measures. Stainless steel finger electrodes (MLT118F, ADInstruments, Colorado Springs, CO, USA) were placed on the index and middle finger pads of the right hand closest to the palm for skin conductivity measures. Rectal temperature, skin temperature, heart rate, SkBF, and skin conductivity were recorded at 1000 Hz and incorporated into an automated digital to analog converter (PowerLab, ADInstruments, Colorado Springs, CO, USA).

Measurement began with a 5‐min seated period at ambient temperature (22.0 ± 1.7°C, 21.8 ± 5.6% RH) to calibrate SkBF and skin conductivity measures before entering the heat chamber for the initial 30‐min exposure period consisting of 5 min seated, 20 min walking, and 5 min seated. Following this 30‐min heat exposure, participants exited the chamber for a nude body weight and an immediate blood draw (blood on days 1 and 7 only). Sustained group participants (SM and SF) spent on average 7.0 ± 1.7 min outside of the heat chamber before returning to finish the final 60 min of their 90‐min heat exposure. Periodic group participants (PM and PF) were permitted to leave the laboratory between their second and third trials of the day (average separation time, 254 ± 34 min). With data only collected during the initial 30‐min testing period on days 1, 4, and 7, all other exposures/sessions were completed without instrumentation. Water consumption was prohibited during the first 30 min of heat exposure but permitted ad libitum at chamber temperature during the final 60 min of heat exposure and when not instrumented. Work (J) during the heat exposures was calculated via the following equation: $\left(\text{Body weight}\right)\times \left(9.81\right)\times \left(\text{vertical distance}\right)$.

### 2.5 Blood sampling and analysis

Blood was collected into a K2E K2EDTA tube (BD, Franklin Lakes, NJ, USA) and inverted 5 times. A whole blood aliquot was removed and collected into a microcuvette (801 microcuvette, Brea, California, USA) and capillary tube. Hemoglobin was immediately measured in an automated microcuvette based system clinically accurate to ±0.5 g·dL^−1^ (Hb 201+, HemoCue, Brea, CA) (Gehring et al., 2002). Capillary tubes were centrifuged (A13, Jouan Incorporated, Winchester, Virginia, USA) at 12,000 RPM for 1 min. Centrifuged capillary tubes were measured in triplicate for hematocrit. The change in plasma volume (∆PV) was calculated from hemoglobin and hematocrit measurements (Dill & Costill, 1974). The remaining whole blood was spun in a benchtop centrifuge (MR22i, Jouan Incorporated, Winchester, Virginia, USA) for 10 min at 10,000 RPM and 4°C.

Individual plasma aliquots were stored at −30°C until analysis of progesterone and estradiol in female participants to confirm that the female groups completed the protocol with a similar ovarian hormone fluctuation for direct comparison. Female participants were of reproductive age (18–35 years); however, eumenorrhea was not verified. Retroactive categorization of binary menstrual cycle phase from progesterone and estradiol values was imprecise due to overlapping phase‐to‐phase hormonal ranges (Gloe et al., 2023) as well as the known hormonal variability between individuals and within a single individual (Baker et al., 2020). Plasma samples collected prior to heat exposure on days 1 and 7 were analyzed in duplicate via enzyme linked immunosorbent assay (ELISA). Commercial kits (DRG International Inc., Springfield, NJ, USA) for progesterone (EIA1561, 0.045 ng·mL^−1^ assay sensitivity) and estradiol (EIA2693, 10.6 pg·mL^−1^ assay sensitivity) were used according to manufacturer protocols. Microplates were automatically washed (1575 Immunowash Plate Washer, Bio‐Rad, Hercules, CA, USA) and read at 450 nm with an additional 620 nm read for background subtraction (VersaMax Microplate Reader, Molecular Devices, San Jose, CA, USA). A standard curve was completed with each microplate and plotted with a 4‐parameter logistic regression.

### 2.6 Sweat rate and sweat composition

Changes in nude body weight during the initial pre to post 30‐min heat exposure session were used to extrapolate hourly sweat rate with the assumption that weight lost was equal to sweat loss. Sweat samples were collected from the forehead with absorbent patches (Suresite 123+, Medline, Northfield, IL, USA) during minutes 5–15 of exercise during the initial 30‐min heat exposure. The forehead was chosen for ease of replication. The forehead was cleaned with alcohol and dried with gauze before absorbent patch placement. Sweat saturated absorbent patches were removed and placed into a 5 mL syringe and compressed with the plunger into a 1.5 mL microcentrifuge tube. Sweat (50 μL) was analyzed in triplicate using ion selective electrodes (LAQUAtwin Ion Meter, Camlab Ltd., Cambridge, United Kingdom, ±10% instrument accuracy) and sampling sheets (Y046, Advantec MFS, Dublin, CA, USA) for sodium and potassium concentration.

### 2.7 Temperature and heart rate

From the initial 30‐min testing period, the final 5 min of walking data (minutes 20–25) were averaged and used in the analysis of rectal temperature, surface temperature, and heart rate to represent the highest heat stress achieved during the measurement window. Standard equations were used to calculate surface temperature as a weighted aggregate of all 3 skin temperature sites (Burton, 1935): $0.50\;\left(\text{chest}\right)+0.14\;\left(\text{forearm}\right)+0.36\;\left(\text{lower}\;\mathrm{leg}\right)$.

### 2.8 Skin blood flow (SkBF) and skin conductivity

SkBF and skin conductivity are functionally related but distinct, since their action is not always parallel (Cramer et al., 2017). SkBF is a relative and regional measure of blood cell movement beneath a reference probe, wherein higher values indicate more blood movement beneath the skin. Skin conductivity is a relative measure of sweat gland activity where greater conductivity suggests greater sweat gland activity (Rosales et al., 2024; Rosales, Powers, et al., 2023; Rosales, Walters, et al., 2023). Skin conductivity is a transient measure complete within the initial 10–15 min of heat stress or exercise (Rosales, Powers, et al., 2023; Rosales, Walters, et al., 2023) and relates to the onset of sweat.

SkBF was recorded on a single channel blood flowmeter (INL191, ADInstruments, Colorado Springs, CO USA) and skin conductivity was recorded on a galvanic skin response amplifier (FE116, ADInstruments, Colorado Springs, CO, USA). SkBF and skin conductivity are expressed relative to the 5‐min ambient temperature calibration period and repeated each testing day. Laser doppler flowmetry surface probes are sensitive to movement artifact which precluded measures while walking. To combat movement artifact, a standardized arm‐propped seating position, low pass 50 Hz filter, and 5‐s smoothing were used during the seated periods of heat exposure (0–5 and 25–30 min). Skin conductivity is expressed in reference to a 40 μS maximal saturation value attainable by the above make and model galvanic skin response amplifier. Skin conductivity markers were quantified as the time to reach 40 μS as well as the rectal and surface temperatures at 40 μS.

### 2.9 Statistical analysis

Differences in body composition and aerobic fitness were analyzed with a one‐way analysis of variance (ANOVA; group). Progesterone and estradiol were analyzed with a two‐way repeated measures ANOVA (female heat exposure schedule × heat exposure day). Differences in temperature (surface, rectal and gastrointestinal), heart rate, sweat rate, sweat composition, and skin conductivity were evaluated using a 3‐way mixed ANOVA (heat exposure schedule × sex × heat exposure day). Differences in SkBF and hematologic markers were evaluated with a 4‐way mixed ANOVA (heat exposure schedule × sex × heat exposure day × pre/post heat exposure). Statistical significance was set at a type I probability error of <5% (*α* < 0.05) with data expressed as mean ± SD. Sphericity was evaluated using Mauchly's test of sphericity and, if violated, corrected using the Huynh‐Feldt procedure. In the event of a significant interaction, a Fisher's Protected Least Significant Difference post‐hoc analysis was used. Statistical analysis was completed with SPSS Version 28 (IBM, Chicago, IL USA). An a priori power analysis using the default settings for a 3‐way mixed ANOVA on G*Power (Heinrich‐Heine‐University, Düsseldorf, Germany) and predicted changes in rectal temperature revealed that 40 participants were required for a power of 0.80. Thus, suggesting adequate power for this *n* = 60 investigation.

## 3 RESULTS

### 3.1 Participant descriptive data

Heat exposure schedule groups (SM, SF, PM, and PF) did not differ in age, weight, height, body surface area, sum of 7 skinfolds, percent body fat, relative $\dot{V}O_{2}$ peak (mL·kg^−1^·min^−1^, mL·kg FFM^−1^·min^−1^), absolute $\dot{V}O_{2}$ peak, or work completed during the heat exposures (*p* > 0.05). Males and females did not differ in age or sum of 7 skinfolds (*p* > 0.05). Males were heavier and taller with a greater body surface area and lower percent body fat than females (*p* < 0.001). Males had a higher relative and absolute $\dot{V}O_{2}$ peak than females and completed more work during the heat exposures (*p* < 0.001). Participant descriptive data are presented in Table 1.

Eight of 30 sustained participants (2 males and 6 females) needed additional rest periods the first 5–6 days that on average totaled 8.4 ± 4.4 min per person during the final 60 min of heat exposure due to nausea, lightheadedness, and/or vomiting. All sustained participants completed the entire walking protocol by day 7. These additional rest periods did not affect the dataset since collection occurred during the initial 30 min of exposure, which every participant completed in compliance with the protocol.

Progesterone was not different between the SF (day 1: 5.5 ± 10.7 ng·mL^−1^; day 7: 1.2 ± 1.0 ng·mL^−1^) and PF (day 1: 3.5 ± 6.5 ng·mL^−1^; day 7: 5.2 ± 8.9 ng·mL^−1^; *p* = 0.624) groups or change from day 1 to 7 in any group (*p* = 0.150). Estradiol was also not different between the SF (day 1: 87.0 ± 98.8 pg·mL^−1^; day 7: 45.9 ± 46.7 pg·mL^−1^) and PF (day 1: 85.7 ± 103.7 pg·mL^−1^; day 7: 49.1 ± 27.8 pg·mL^−1^; *p* = 0.968) groups. However, estradiol did decrease from day 1 to day 7 in both groups (*p* = 0.026). This between group and time consistency allows for direct comparison of the female groups (individual responses are displayed in Figure S1a,b).

### 3.2 Surface temperature

**FIGURE 2:**
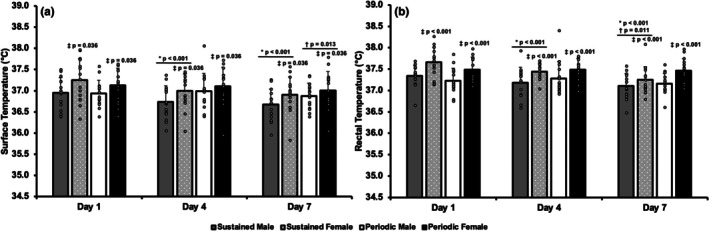
Mean and individual surface (a) and rectal (b) temperatures while walking during minutes 20–25 of heat exposure on Days 1, 4, and 7 of two heat acclimation regimens. *p < 0.05 from day 1 within a group, ^†^ *p* < 0.05 from day 4 within a group, ^‡^ *p* < 0.05 from males. Data presented as mean ± SD.

A significant ANOVA interaction for schedule by heat exposure day was observed in mean surface temperature (*p* < 0.001). In the sustained group, mean surface temperature decreased from day 1 on days 4 (*p* < 0.001) and 7 (*p* < 0.001) but did not further decrease from day 4 to 7 (*p* = 0.090). In the periodic group, mean surface temperature did not change from day 1 to days 4 (*p* = 0.726) or 7 (*p* = 0.109), but day 4 was higher than day 7 (*p* = 0.013). When mean surface temperature on days 1, 4, and 7 is directly compared between exposure schedules, there is no difference (*p* > 0.05). A significant ANOVA main effect for sex was observed in mean surface temperature (*p* = 0.036). Mean surface temperatures were lower overall in males than females during all heat exposures. However, when the rise in surface temperature from baseline is examined, sex differences are diminished (*p* = 0.647, data not shown). Mean surface temperature and individual responses are presented in Figure 2a. Mean and individual chest, forearm, and calf skin temperatures are displayed in Figure S2a–c.

### 3.3 Rectal temperature

A significant ANOVA interaction for schedule by heat exposure day was observed in mean rectal temperature (*p* < 0.001). Mean rectal temperature in the sustained group decreased from day 1 to days 4 and 7 (*p* < 0.001) with a further decrease from day 4 to 7 (*p* = 0.011). Mean rectal temperature in the periodic group did not exhibit any changes from day 1 to days 4 (*p* = 0.537) and 7 (*p* = 0.364) or from day 4 to 7 (*p* = 0.135). When mean rectal temperature on days 1, 4, and 7 is directly compared between exposure schedules, there is no difference (*p* > 0.05). A significant ANOVA main effect for sex was observed in mean rectal temperature (*p* < 0.001). Mean rectal temperature was lower in males than females during all heat exposures. However, when the rise in rectal temperature from baseline is examined, sex differences are diminished (*p* = 0.957, data not shown). Mean rectal temperature and individual responses are presented in Figure 2b. Body temperature, an aggregate calculation of surface and rectal temperatures, mirrored changes in rectal temperature (Figure S3).

### 3.4 Gastrointestinal temperature

A significant ANOVA main effect in peak gastrointestinal temperature was observed for exposure schedule (*p* < 0.001). The sustained group (grand mean, 38.6 ± 0.5°C) experienced a higher peak gastrointestinal temperature than the periodic group (grand mean, 38.2 ± 0.3°C). There was not a significant ANOVA main effect in peak gastrointestinal temperature for sex (*p* = 0.848). Peak gastrointestinal temperatures were similar in males and females (male grand mean, 38.4 ± 0.5°C; female grand mean, 38.4 ± 0.4°C). A significant ANOVA main effect was observed for heat exposure day (*p* < 0.001). Peak gastrointestinal temperature did not change from day 1 (38.5 ± 0.5°C) to day 4 (38.5 ± 0.5°C, *p* = 0.365), but day 1 was higher than day 7 (38.3 ± 0.4°C, *p* < 0.001). Peak gastrointestinal temperature decreased from day 4 to 7 (*p* = 0.002).

### 3.5 Heart rate

**FIGURE 3:**
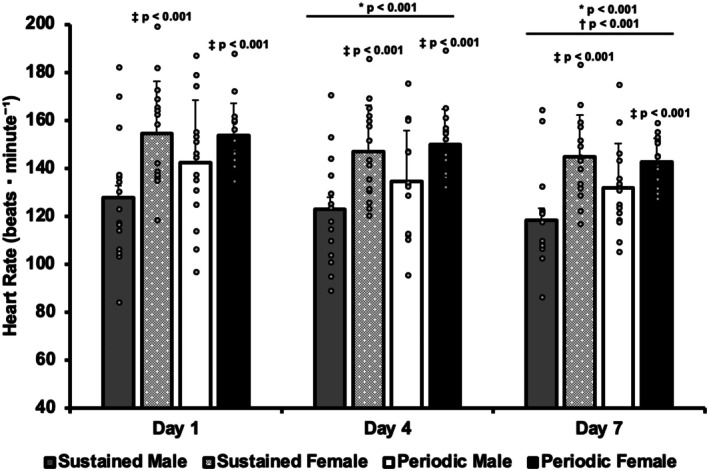
Mean and individual heart rate responses while walking during minutes 20–25 of heat exposure on days 1, 4, and 7 of two 7‐day heat acclimation regimens. *p < 0.05 from day 1, ^†^ *p* < 0.05 from day 4, ^‡^ *p* < 0.05 from males. Data presented as mean ± SD.

No significant ANOVA main effect was observed for exposure schedule in mean heart rate (*p* = 0.188). Mean heart rate did not differ between the sustained or periodic groups. Significant ANOVA main effects for day (*p* < 0.001) and sex (*p* < 0.001) were observed. Mean heart rate decreased from day 1 to day 4 and 7 of heat exposure (*p* < 0.001), with a further decrease from day 4 to 7 (*p* < 0.001). Mean heart rate was lower in males than females during each heat exposure (*p* < 0.001). Mean heart rate and individual responses are presented in Figure 3.

### 3.6 Sweat rate, skin conductivity, and sweat composition

There was not a significant ANOVA main effect for exposure schedule in absolute (*p* = 0.613) and relative sweat rates (*p* = 0.482). The periodic and sustained groups did not differ in sweat rate. There was a significant ANOVA main effect for sex in absolute (*p* < 0.001) and relative (*p* = 0.022) sweat rates. Specifically, absolute and relative sweat rates were higher in males than females. There was no significant ANOVA main effect for heat exposure day in absolute (*p* = 0.655) and relative (*p* = 0.581) sweat rates. Sweat rate did not change during the 7‐day acclimation period. Sweat rate is presented in Table 2.

**TABLE 2:** Sweat metrics during Days 1, 4, and 7 of two 7‐day heat acclimation regimens.

	Day 1	Day 4	Day 7
Sweat rate (L·h^−1^)			
Sustained male	0.91 ± 0.41	0.94 ± 0.39	0.90 ± 0.44
Sustained female	0.63 ± 0.2^§^	0.66 ± 0.24^§^	0.64 ± 0.24^§^
Periodic male	0.96 ± 0.35	0.99 ± 0.39	0.96 ± 0.42
Periodic female	0.68 ± 0.15^§^	0.66 ± 0.21^§^	0.68 ± 0.28^§^
Sweat Rate (L·h^−1^·m^2(−1)^)			
Sustained male	0.44 ± 0.18	0.46 ± 0.16	0.44 ± 0.18
Sustained female	0.36 ± 0.10^§^	0.38 ± 0.12^§^	0.38 ± 0.13^§^
Periodic male	0.48 ± 0.14	0.49 ± 0.16	0.47 ± 0.18
Periodic female	0.40 ± 0.09^§^	0.38 ± 0.11^§^	0.39 ± 0.15^§^
Time to 40 μS (s)			
Sustained male	988 ± 170	1038 ± 305	1038 ± 324
Sustained female	1062 ± 384	1121 ± 335	1017 ± 277
Periodic male	978 ± 233	987 ± 222	1017 ± 200
Periodic female	1039 ± 327	991 ± 320	1016 ± 310
Surface temperature at 40 μS (°C)			
Sustained male	36.3 ± 0.6	35.9 ± 0.9	35.9 ± 0.8^†^
Sustained female	36.5 ± 0.6	36.4 ± 0.6	36.3 ± 0.7^†^
Periodic male	36.0 ± 0.7	36.3 ± 0.4	36.2 ± 0.5
Periodic female	36.2 ± 0.7	36.2 ± 0.7	36.2 ± 0.7
Rectal temperature at 40 μS (°C)			
Sustained male	37.2 ± 0.2	37.1 ± 0.3^†^	37.0 ± 0.4^†^
Sustained female	37.5 ± 0.4^§^	37.3 ± 0.2^†^ ^,^ ^§^	37.3 ± 0.6^†^ ^,^ ^§^
Periodic male	37.1 ± 0.4	37.0 ± 0.4^†^	36.9 ± 0.3^†^
Periodic female	37.3 ± 0.4^§^	37.2 ± 0.3^†^ ^,^ ^§^	37.3 ± 0.3^†^ ^,^ ^§^
Sodium (mmol·L^−1^)			
Sustained male	66.0 ± 20.8	50.3 ± 18.9^†^	43.9 ± 17.7^†^ ^,^ ^‡^
Sustained female	67.8 ± 23.0	56.7 ± 21.6^†^	51.8 ± 21.4^†^ ^,^ ^‡^
Periodic male	76.6 ± 16.2	72.6 ± 21.5* ^,^ ^†^	56.8 ± 16.1^†^ ^,^ ^‡^
Periodic female	70.5 ± 21.4	62.7 ± 23.4* ^,^ ^†^	53.7 ± 21.5^†^ ^,^ ^‡^
Potassium (mmol·L^−1^)			
Sustained male	7.1 ± 2.2	7.0 ± 1.5	6.8 ± 0.9
Sustained female	8.4 ± 1.7^§^	8.1 ± 1.7^§^	8.3 ± 1.6^§^
Periodic male	6.2 ± 1.3	6.6 ± 1.1	6.9 ± 1.7
Periodic female	8.2 ± 1.7^§^	7.9 ± 0.8^§^	8.2 ± 1.2^§^

*Note*: Data presented as mean ± SD.

* *p* < 0.05 from sustained within a day.

^†^ *p* < 0.05 from Day 1.

^‡^ *p* < 0.05 from Day 4.

^§^ *p* < 0.05 from males.

There was not a significant ANOVA main effect in skin conductivity time to 40 μS for exposure schedule (*p* = 0.569), sex (*p* = 0.628), or day (*p* = 0.799). Time to 40 μS was not different between the periodic and sustained groups or between males and females. Time to 40 μS did not change during the 7‐day acclimation period. Time to 40 μS is presented in Table 2.

A significant ANOVA interaction was observed in surface temperature at a skin conductivity of 40 μS for exposure schedule (*p* = 0.032). The sustained groups' surface temperature at 40 μS did not change from day 1 to day 4 (*p* = 0.057) but was lower when day 1 was compared to day 7 (*p* = 0.015). There was no change in surface temperature at 40 μS in the sustained group from day 4 to day 7 (*p* = 0.322). Surface temperature at 40 μS in the periodic group did not change from day 1 to days 4 (*p* = 0.335) and 7 (*p* = 0.422) or change from day 4 to 7 (*p* = 1.00). Surface temperature at 40 μS was not different between the sustained and periodic groups when directly compared on days 1, 4, and 7 (*p* > 0.05). No significant ANOVA main effect was observed in surface temperature at 40 μS for sex (*p* = 0.206). Surface temperature at 40 μS was not different between males and females. Surface temperature at 40 μS is presented in Table 2.

There was not a significant ANOVA main effect in rectal temperature at a skin conductivity of 40 μS for exposure schedule (*p* = 0.536). The periodic and sustained groups did not have differing rectal temperatures at 40 μS. A significant ANOVA main effect in rectal temperature at 40 μS was observed for sex (*p* < 0.001) and day (*p* = 0.008). Males had a lower rectal temperature at 40 μS than females. Rectal temperature at 40 μS decreased from day 1 to day 4 (*p* = 0.012) and 7 (*p* = 0.004) of heat exposure but there was no further decrease from day 4 to 7 (*p* = 0.487). Rectal temperature at 40 μS is presented in Table 2.

A significant ANOVA interaction was observed in sweat sodium concentration for exposure schedule (*p* = 0.034). Sweat sodium concentration in the sustained and periodic groups decreased from day 1 to days 4 (*p* < 0.001, *p* = 0.012, respectively) and 7 (*p* < 0.001, *p* < 0.001, respectively) with further decreases from day 4 to 7 (*p* = 0.006, *p* < 0.001, respectively). When directly comparing schedules, sweat sodium concentration was not different on day 1 (SM, SF, PM, and PF; *p* = 0.217), was lower in the sustained groups on day 4 (*p* = 0.013), and was not different on day 7 (*p* = 0.141). There was not a significant ANOVA main effect in sweat sodium concentration for sex (*p* = 0.920). Males and females did not have differing sweat sodium concentrations. Sweat sodium concentration is presented in Table 2.

There was no significant ANOVA main effect in sweat potassium concentration for exposure schedule (*p* = 0.354) or heat exposure day (*p* = 0.724). The periodic and sustained groups did not differ in sweat potassium concentration, nor did sweat potassium concentration change during the 7‐day acclimation protocol. A significant ANOVA main effect for sweat potassium concentration was observed for sex (*p* < 0.001). Sweat potassium concentration was more dilute in males than females. Sweat potassium concentration is presented in Table 2.

### 3.7 Skin blood flow (SkBF)

**FIGURE 4:**
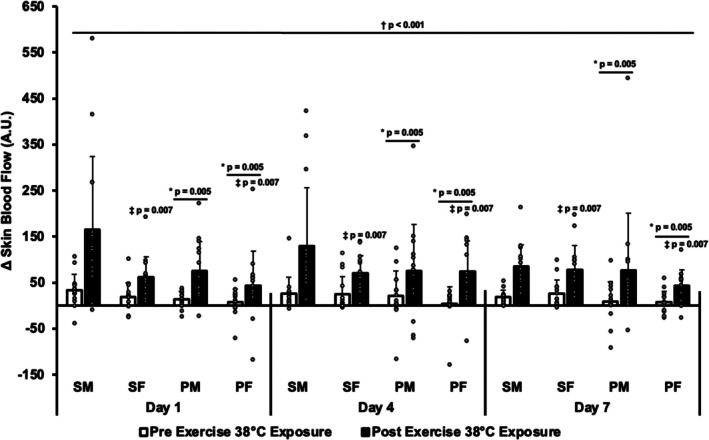
Mean relative change (∆) and individual responses in skin blood flow upon entering the heat chamber (pre‐exercise 38°C exposure) and prior to exiting (post exercise 38°C exposure) on days 1, 4, and 7 of two 7‐day heat acclimation regimens. PF, periodic female; PM, periodic male; SF, sustained female; SM, sustained male. *p < 0.05 from sustained pre and post exercise 38°C exposure, ^†^ *p* < 0.05 from pre exercise 38°C exposure, ^‡^ *p* < 0.05 from males post exercise 38°C exposure. Data presented as mean ± SD.

A significant ANOVA main effect was observed in SkBF for exposure schedule (*p* = 0.005). The sustained groups had higher SkBF than the periodic groups during each heat exposure. There was a significant ANOVA interaction in SkBF for pre/post walking and sex (*p* = 0.016). When entering the heat chamber, SkBF was similar between males and females (*p* = 0.299). Both sexes increased SkBF from pre‐ to post‐30 min heat exposure/walking (*p* < 0.001), but males had an incrementally higher SkBF than females (*p* = 0.007). There was not a significant ANOVA main effect in SkBF for heat exposure day (*p* = 0.457). SkBF did not change during the 7‐day acclimation period. Mean SkBF and individual responses are presented in Figure 4.

### 3.8 Hemoglobin, hematocrit, and relative change in plasma volume (∆PV)

There was not a significant ANOVA main effect in hemoglobin for exposure schedule (*p* = 0.552). Hemoglobin was not different between the sustained and periodic groups. A significant ANOVA main effect in hemoglobin for sex was observed (*p* < 0.001). Males had higher hemoglobin than females. A significant ANOVA main effect in hemoglobin was observed for pre‐ to post‐ 30‐min heat exposure (*p* < 0.001). Thirty minutes of heat exposure concentrated hemoglobin. A significant ANOVA main effect was observed in hemoglobin for heat exposure day (*p* < 0.001). Hemoglobin was diluted during the 7‐day acclimation period. Hemoglobin is presented in Table 3.

**TABLE 3:** Hematic data pre‐ and post‐ 30‐min heat exposure on days 1 and 7 of two 7‐day heat acclimation regimens.

	Day 1		Day 7	
	Pre heat exposure	Post heat exposure	Pre heat exposure	Post heat exposure
Hemoglobin (g·dL^−1^)				
Sustained male	15.9 ± 1.1	16.4 ± 1.3*	15.4 ± 1.1^†^	16.0 ± 1.0* ^,^ ^†^
Sustained female	13.7 ± 1^‡^	14.4 ± 1.1* ^,^ ^‡^	13.6 ± 1^†^ ^,^ ^‡^	13.9 ± 0.9* ^,^ ^†^ ^,^ ^‡^
Periodic male	16.0 ± 0.9	16.4 ± 0.8*	15.3 ± 0.7^†^	15.7 ± 0.8* ^,^ ^†^
Periodic female	14.3 ± 1.0^‡^	14.7 ± 1.0* ^,^ ^‡^	13.6 ± 0.7^†^ ^,^ ^‡^	14.0 ± 0.8* ^,^ ^†^ ^,^ ^‡^
Hematocrit (%)				
Sustained male	51.2 ± 4.1	51.7 ± 5.0*	49.6 ± 3.3^†^	49.8 ± 2.8* ^,^ ^†^
Sustained female	45.1 ± 3.0^‡^	46.7 ± 3.1* ^,^ ^‡^	44.2 ± 3.5^†^ ^,^ ^‡^	45.0 ± 3.1* ^,^ ^†^ ^,^ ^‡^
Periodic male	51.3 ± 2.3	52.4 ± 2.3*	49.8 ± 3.3^†^	50.2 ± 2.7* ^,^ ^†^
Periodic female	45.9 ± 3.1^‡^	47.1 ± 2.7* ^,^ ^‡^	44.4 ± 2.2^†^ ^,^ ^‡^	44.9 ± 2.3* ^,^ ^†^ ^,^ ^‡^
∆ Plasma Volume (%)	Set to 0		Set to 0	
Sustained male	—	−3.6 ± 5.0	—	−4.0 ± 6.6
Sustained female	—	−7.4 ± 6.9	—	−3.4 ± 6.3
Periodic male	—	−4.5 ± 6.9	—	−3.6 ± 5.8
Periodic female	—	−5.0 ± 7.7	—	−3.3 ± 5.4

	Pre heat exposure (set to 0)	Pre heat exposure
∆ plasma volume (%)		
Sustained male	—	7.7 ± 12.1^†^
Sustained female	—	2.7 ± 9.4^†^
Periodic male	—	9.1 ± 14.6^†^
Periodic female	—	8.0 ± 9.5^†^

*Note*: Data presented as mean ± SD.

* *p* < 0.05 from pre heat exposure within a day.

^†^ *p* < 0.05 from Day 1.

^‡^ *p* < 0.05 from Males.

There was not a significant ANOVA main effect in hematocrit for exposure schedule (*p* = 0.624). Hematocrit was not different between the sustained and periodic groups. A significant ANOVA main effect in hematocrit was observed for sex (*p* < 0.001). Males had higher hematocrit than females. A significant ANOVA interaction was observed in hematocrit for pre/post 30‐min exposure and heat exposure day (*p* = 0.037). On days 1 and 7, hematocrit increased due to heat exposure (*p* < 0.001). Hematocrit on day 1 was higher than day 7, both pre and post 30‐min exposure (*p* < 0.001). Hematocrit is presented in Table 3.

There was not a significant ANOVA main effect in ∆PV due to the 30‐min exposure for group (*p* = 0.676), sex (*p* = 0.583), or heat exposure day (*p* = 0.135). The ∆PV due to the 30‐min exposure did not differ between the sustained and periodic groups or between males and females. The ∆PV due to the 30‐min exposure did not change during the 7‐day acclimation period. There was not a significant ANOVA main effect in ∆PV from day 1 to 7 for group (*p* = 0.271) or sex (*p* = 0.316). The ∆PV from day 1 to 7 did not differ between the sustained and periodic groups or between males and females. A significant ANOVA main effect was observed in ∆PV from day 1 to 7 for heat exposure day (*p* < 0.001). A ∆PV increase was observed from day 1 to 7. ∆PV is presented in Table 3.

## 4 DISCUSSION

The purpose of this study was to investigate the effectiveness of two differing 7‐day heat acclimation regimens while also measuring sex‐specific adaptations. The sustained protocol (7 visits, 1 × 90‐min daily heat exposure session) was designed to follow a conventional heat acclimation approach (Bean et al., 1943; Cohen & Gisolfi, 1982; Duncan, 1966; Frye & Kamon, 1981; Nadel et al., 1974; Robinson et al., 1943; Wyndham et al., 1965) of prolonged daily heat exposures (Sawka et al., 2003; Centers for Disease Control and Prevention (CDC), 2023; Occupational Health and Safety Administration (OSHA), 2025; Eifling et al., 2024; Périard et al., 2015). Conversely, the periodic protocol (21 visits, 3 × 30‐min daily heat exposure sessions) was designed to permit temporary thermal recovery (minimum 3 h recovery) by utilizing shorter individual sessions. The periodic protocol is not as effective at inducing heat acclimation as the sustained protocol. Matching ambient temperature, cumulative duration, and exercise workload without specifically considering the heat exposure schedule will limit heat adaptations. The periodic group's acclimation outcomes do not support recommendations advocating for shorter individual sessions (Sawka et al., 2003; Centers for Disease Control and Prevention (CDC), 2023). Interestingly, sex differences were consistent and unchanged even as acclimation progressed across both groups. The present data do not corroborate any postulation that females are more susceptible to exertional heat related illnesses compared to males (Alele et al., 2020; Kazman et al., 2015; van Steen et al., 2019) following acclimation.

Effective heat acclimation regimens are expected to induce thermoregulatory changes resulting in lower steady state maintenance of core temperature (Barnett & Maughan, 1993; Bean et al., 1943; Cohen & Gisolfi, 1982; Duncan, 1966; Frye & Kamon, 1981; Nadel et al., 1974; Robinson et al., 1943; Wyndham et al., 1965), skin temperature (Barnett & Maughan, 1993; Cohen & Gisolfi, 1982; Duncan, 1966; Frye & Kamon, 1981; Robinson et al., 1943), and heart rate (Barnett & Maughan, 1993; Bean et al., 1943; Cohen & Gisolfi, 1982; Duncan, 1966; Frye & Kamon, 1981; Robinson et al., 1943; Wyndham et al., 1965). Although these adaptations would be substantiated during a heat tolerance test, the design required that thermoregulatory measurements occur within the first 30 min of the 90‐min exposure for unconfounded group comparison. This is an important and appropriate aspect of the study design to protect internal validity and avoid contaminating the periodic group since a single 60–120 min exposure can begin inducing acclimation (Bean et al., 1943). Despite reducing the primary study markers of heat acclimation to the first 30 min, both groups demonstrated measurable thermoregulatory differences. The environment (38°C, 60% relative humidity) and exercise (1.6 m·s^−1^, 5% grade; 6.1 METs) combination accentuated thermoregulatory differences that should persist during a longer thermal challenge. In acknowledging that a 30‐min measurement period would exclude peak heat stress, telemetry pills were used to extend heat stress examination for the full 90‐min. Simplified tabular conclusions for each experimental grouping are presented within Table 4.

**TABLE 4:** Tabular summary of conclusions from both 7‐day heat acclimation regimens.

	Sustained male (SM)	Sustained female (SF)	Periodic male (PM)	Periodic female (PF)
Rectal temperature^a^	↓	↓	⎯	⎯
Surface temperature (3 skin site aggregate)^a^	↓	↓	↓	↓
Skin conductivity				
Time to 40 μS	⎯	⎯	⎯	⎯
Rectal temperature at 40 μS^a^	↓	↓	↓	↓
Surface temperature at 40 μS	↓	↓	⎯	⎯
Sweat				
Absolute rate^a^	⎯	⎯	⎯	⎯
Relative rate^a^	⎯	⎯	⎯	⎯
Sodium concentration	↓	↓	↓	↓
Potassium concentration^a^	⎯	⎯	⎯	⎯
Heart rate^a^	↓	↓	↓	↓
Skin blood flow^a^	⎯	⎯	⎯	⎯
Gastrointestinal Temperature	↓	↓	↓	↓
∆ plasma volume	↑	↑	↑	↑

*Note*: The ↓, ↑, and ⎯ symbols indicate increase, decrease, or no change, respectively, during 7 days of heat exposure.

^a^ Indicates a sex difference.

Current recommendations attesting that heat acclimation protocols can be segmented into smaller sessions (Sawka et al., 2003; Centers for Disease Control and Prevention (CDC), 2023) may inadequately perturb core temperature if the ambient environment and/or workload are not intensified. A core temperature of 38.5°C is an established threshold for potent heat stress (Amorim et al., 2008; Gibson et al., 2014) and is oftentimes the numerical target of isothermal heat acclimation strategies (Périard et al., 2015). Over a 9 day period, heat acclimating with single bouts of heat stress (1 × 100 min) incurs a greater magnitude of adaptation than two separate bouts of duration‐matched heat stress (2 × 50 min) (Lind & Bass, 1963). To succeed this previous intermittent design (Lind & Bass, 1963), the environmental temperature was set to nearly uncompensable levels (Eichna et al., 1945; National Weather Service (NWS), 2025) at a 4°C higher wet bulb temperature and occurred with an additional 2.8 METs. Only the sustained group's peak gastrointestinal temperature breached 38.5°C (38.6 ± 0.5°C vs. 38.2 ± 0.3°C), on average achieving a more potent heat stress (Amorim et al., 2008; Gibson et al., 2014). The sustained group demonstrated more complete acclimation. Mainly, accelerated sweat sodium concentration dilution, higher SkBF, decreased rectal temperature, earlier decrease in surface temperature, and decreased surface temperature at 40 μS. Thus, a critical determinant of heat acclimation is a sustained rise in core temperature regardless of whether environmental conditions, cumulative duration, and exercise workload are matched, corroborating conclusions made in 1963 (Lind & Bass, 1963).

The slighter and intermittent perturbations of rectal/gastrointestinal and surface temperatures allowed the periodic group to experience select heat adaptations: plasma volume expansion, sweating at a lower rectal temperature, and a decreased heart rate. Skin thermoreception and surface temperature increases (Tansey & Johnson, 2015) additionally promote heat adaptations by engaging heat dissipation mechanisms. The periodic approach as presented here, in isolation, may not optimize heat adaptations but could be a pragmatic strategy as lead in/preparation for prolonged heat acclimation regimens. Value in this practice can be extrapolated from the difficulty many sustained participants experienced. Eight sustained participants required additional rest periods during the first 5–6 days within the final 60‐min of exposure due to nausea, lightheadedness, and/or vomiting. Those requiring additional rest were largely female (six participants), but an exact consensus cannot be made from this observation. Conversely, all periodic participants completed sessions without reported or observed adverse reactions. Heat stress symptoms are attributable to the continuous heat load and not merely identical overall duration. Shorter sessions could minimize these symptoms prior to a conventional protocol.

Contrary to the common catalog of heat acclimation markers (Table 4) is that sweat rate (whole body, relative to body surface area) is unchanged in all groups. Steady state sweat output is gradually achieved after 20–30 min of heat stress (Baker, 2017). While the 30‐min measurement period permitted equitable group comparisons, extrapolating hourly sweat rate from shorter measurement intervals may conceal changes. Sweat gland adaptations indicative of heat acclimation were evident based on skin conductivity measures in the sustained group and sweat sodium dilution with both schedules. Skin conductivity, a metric regarded as similar to the onset of sweat (Rosales, Powers, et al., 2023; Rosales, Walters, et al., 2023) improved due to acclimation in the sustained group. Skin conductivity is a transient marker of sweat gland activity (first ~10–15 min of heat stress) and does not provide information regarding steady state sweat output. Sweat sodium concentration persists across a range of sweat rates (Allan & Wilson, 1971; Buono et al., 2007) and were sweat rate to increase after steady state output, this adaptation might remain.

The final aspect of this work addressed thermoregulatory sex‐differences and if heat acclimation is influenced. Female physiology purportedly increases susceptibility for exertional heat‐related illnesses compared to males (Alele et al., 2020; Kazman et al., 2015; van Steen et al., 2019). However, females do acquire heat adaptations for acclimation (Cohen & Gisolfi, 1982; Fein et al., 1975; Frye & Kamon, 1981; Giersch et al., 2025; Sawka et al., 1983; Shapiro et al., 1980; Weinman et al., 1967; Wyndham et al., 1965). When males (Slivka et al., 2021) and females (McGlynn et al., 2022) are acclimated with similar regimens, molecular signaling outcomes can even favor female physiology. The sex‐differences observed here presented upon initial exposure and persisted through the final exposure as elevated rectal/gastrointestinal temperature, surface temperature, and heart rate. Some aspects of these sex‐differences are explained by ovarian hormone circulation, fitness, and anthropometrics. Progesterone is thermogenic in the luteal phase compared to the follicular phase, increasing basal body temperature (+0.3–0.7°C) (Baker et al., 2020). Independent of the menstrual cycle, anthropometrics (higher percent body fat) and aerobic fitness (less fitness) can limit heat dissipation. Although female participants had greater percent body fat and completed each trial at a greater proportion of their $\dot{V}O_{2}$ peak, the rise in rectal temperature from baseline was similar between sexes. Female participants only began the heat exposure warmer, aligning with progesterone's thermogenic effect. Moreover, irrespective of ovarian hormone influence on basal body temperature (Ley et al., 2023), peak gastrointestinal temperature was similar between sexes. If core temperature differs by sex during the early stages of heat stress, these differences can lessen as a thermal challenge progresses.

Exercising in the heat with freely available fluids does not always elicit meaningful sex‐differences in fluid balance, sweat rate, and core temperature (Notley et al., 2022). To thermoregulate and manage heat stress similar to males, some speculate from surface temperature changes that female‐specific adaptations in SkBF drive heat dissipation (McGlynn et al., 2022; Weinman et al., 1967; Wyndham et al., 1965) since female sweat rate does not always change with heat acclimation (Giersch et al., 2025; McGlynn et al., 2022; Sawka et al., 1983; Shapiro et al., 1980; Weinman et al., 1967). Potential female‐specific SkBF adjustments are evident during passive heating, wherein the luteal phase is marked by higher SkBF compared to the follicular phase (Bartelink et al., 1990; Petrofsky et al., 2017). Here, females not only had a lower sweat rate than males but also a lower SkBF. Changes in plasma volume indicative of a greater body water reservoir to sweat and heat dissipate were comparable, with males only possessing higher hemoglobin and hematocrit than females. Females and males commenced sweating after a similar amount of time in the heat (time to 40 μS), with the females occurring at higher rectal temperatures than males. Whole body sweat rate differs by sex but sweat sensitivity to a heat stimulus (skin conductivity) may not. Interestingly, females had a higher sweat potassium concentration, which is an inconsistently measured sex‐difference (Lara et al., 2016).

Binary menstrual phase categorization attempts from progesterone and estradiol values were imprecise due to overlapping phase‐to‐phase hormonal ranges (Gloe et al., 2023). There was also considerable hormonal standard deviation (Figure S1) due to known variability between individuals and even in a single individual (Baker et al., 2020). Ovarian‐hormone mediated core temperature differences during thermal stresses are variable depending on the exact experimental parameters (Christison et al., 2025; Freemas et al., 2023). Ovarian‐hormones fluctuate beyond simplified binary classification and thermoregulatory consequences may not be reducible to two phases. Importantly, the female groups could be compared due to statistically similar ovarian hormone fluctuation. This may not mean the female groups were in the same binary phase (follicular or luteal) but rather indicates that hormonal mediators related to thermoregulation were similar. Small basal body temperature elevations seem less consequential under the combined stressors of exercise and heat and are likely relieved by adequate heat dissipation mechanisms and behavioral decisions.

## 5 CONCLUSIONS

The purpose of this investigation was to examine heat acclimation progression during two varying, but duration and absolute workload‐matched, 7‐day heat acclimation strategies: sustained (7 visits, 1 × 90‐min daily heat exposure session) and periodic (21 visits, 3 × 30‐min daily heat exposure sessions). Matching ambient temperature, duration, and exercise workload between heat acclimation regimens may not result in complete or optimized heat adaptations. Three daily periodic 30‐min sessions are not as effective as one daily 90‐min sustained session at inducing heat adaptations. Further, these data suggest that thermoregulatory sex‐differences are present before and persist throughout heat acclimation. These findings are displayed as simplified tabular conclusions in Table 4.

## AUTHOR CONTRIBUTIONS

DRS and BCR conceived and designed the research. AMR, JLM, ACE, and ALH acquired data. AMR, JLM, and ACE analyzed the data. AMR and DRS interpreted the data. AMR prepared figures and drafted the manuscript. All authors edited, revised, and approved the final manuscript version.

## FUNDING INFORMATION

This research was supported by the United States Department of Defense, Department of the Air Force (FA8650‐19‐C6124).

## CONFLICT OF INTEREST STATEMENT

The authors have no conflicts of interest, financial or otherwise, to declare.

## Supporting information


Figure S1.

Figure S2.

Figure S3.

## Data Availability

Data has not been placed into any public repository but may be available from the corresponding author upon request.
