# Supplementary material for: Comparison of heat acclimation after once daily and thrice daily heat exposures in healthy adults
Source: Physiol Rep. 2026 Feb 26;14(4):e70796. doi: 10.14814/phy2.70796 (PMC12946464; doi:10.14814/phy2.70796)
Supplement: Supplementary file 1 — Figure S1. [file PHY2-14-e70796-s003.pdf]

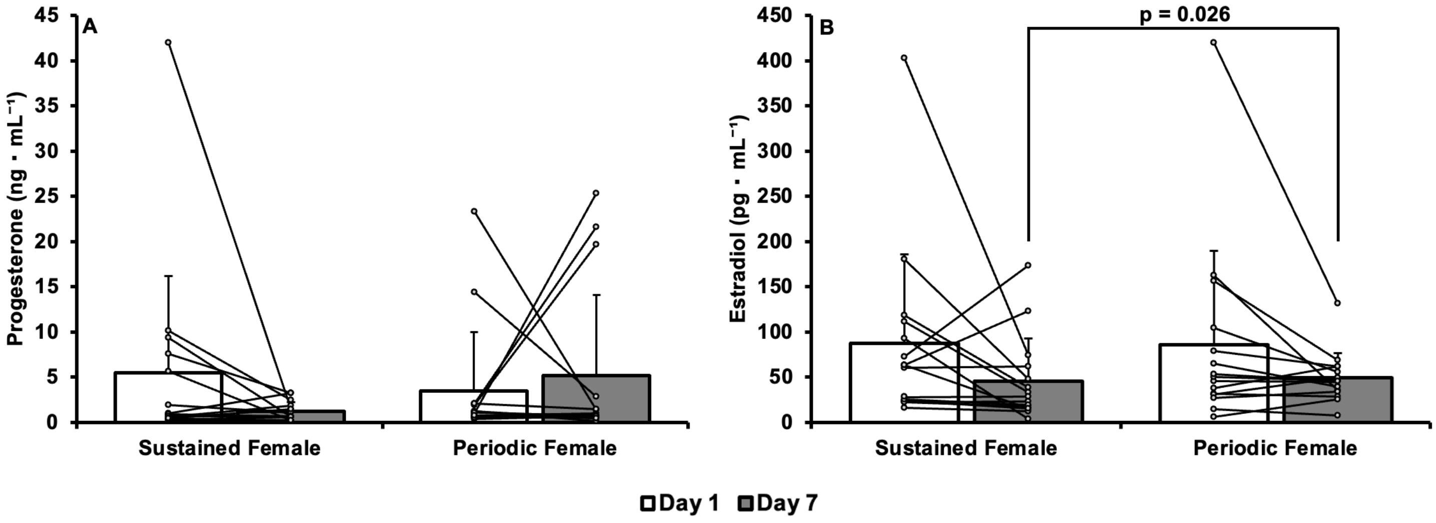

Supplemental Figure 1: Mean and individual ovarian hormone fluctuation (Progesterone, A; Estradiol, B) prior to heat exposure on Days 1 and 7 in the two female heat exposure schedule groups. * $p < 0.05$ from day 1 within each group. Data presented as mean ± SD
