# Supplementary material for: Comparison of heat acclimation after once daily and thrice daily heat exposures in healthy adults
Source: Physiol Rep. 2026 Feb 26;14(4):e70796. doi: 10.14814/phy2.70796 (PMC12946464; doi:10.14814/phy2.70796)
Supplement: Supplementary file 2 — Figure S2. [file PHY2-14-e70796-s001.pdf]

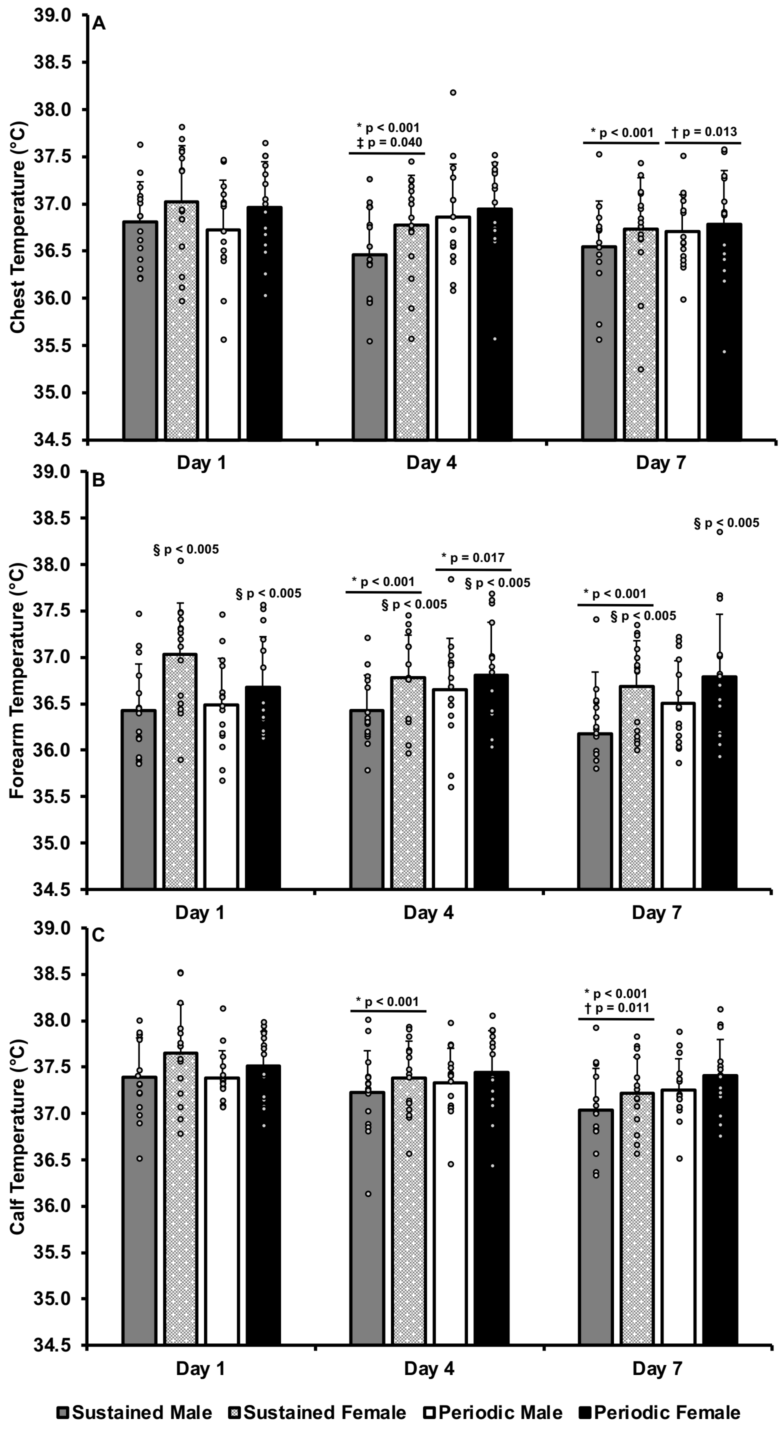

Supplemental Figure 2: Mean and individual skin temperature (Chest, A; Forearm, B; Calf, C) responses while walking during minutes 20-25 of heat exposure on days 1, 4, and 7 of two 7-day heat acclimation regimens. * $p < 0.05$ from day 1 within a group, † $p < 0.05$ from day 4 within a group, ‡ $p < 0.05$ between groups within a day, § $p < 0.05$ from males. Data presented as mean ± SD.
