# Supplementary material for: Comparison of heat acclimation after once daily and thrice daily heat exposures in healthy adults
Source: Physiol Rep. 2026 Feb 26;14(4):e70796. doi: 10.14814/phy2.70796 (PMC12946464; doi:10.14814/phy2.70796)
Supplement: Supplementary file 3 — Figure S3. [file PHY2-14-e70796-s002.pdf]

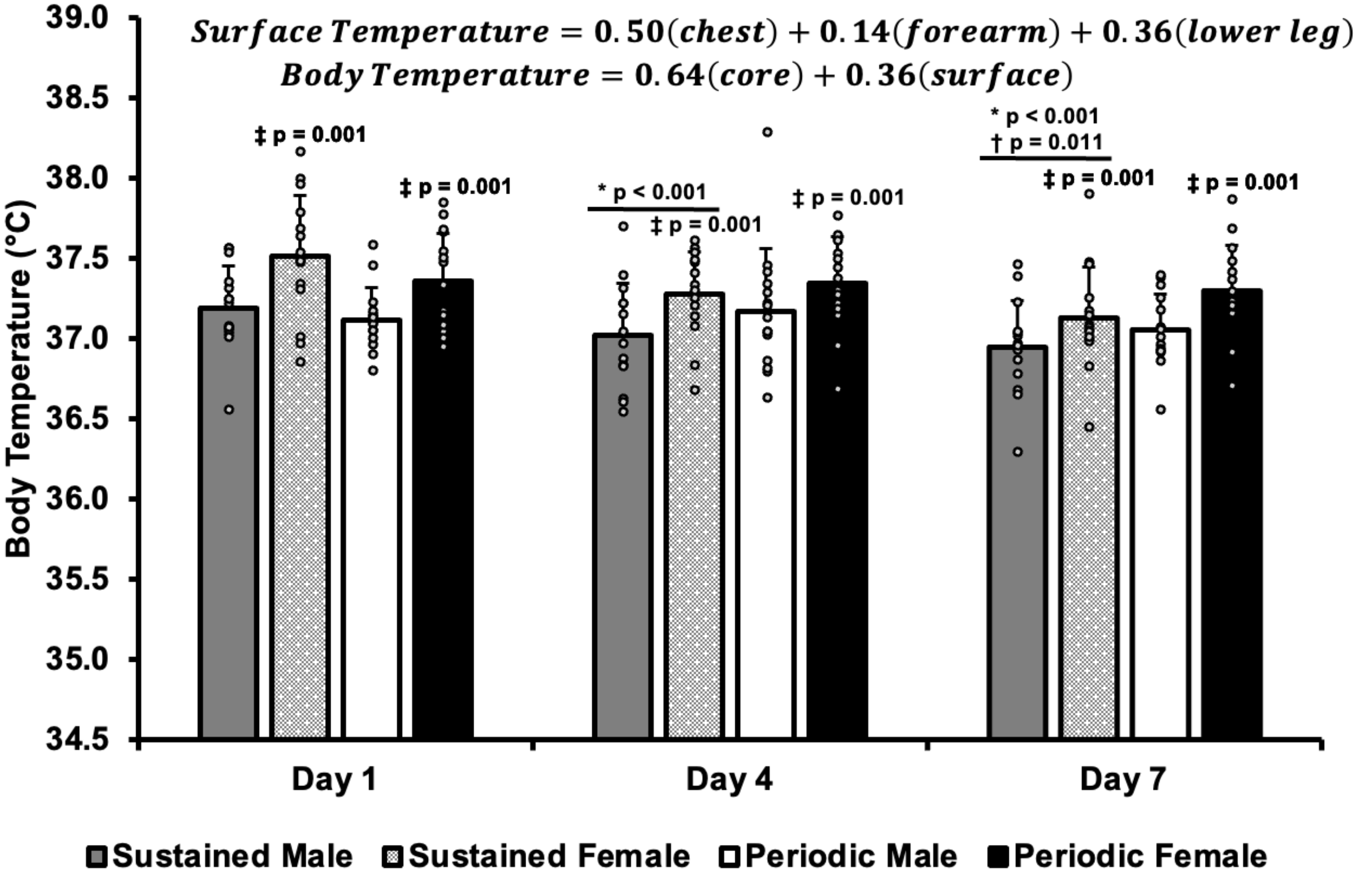

Supplemental Figure 3: Mean and individual body temperature while walking during minutes 20-25 of heat exposure on Days 1, 4, and 7 of two heat acclimation regimens. * $p < 0.05$ from day 1 within a group, † $p < 0.05$ from day 4 within a group, ‡ $p < 0.05$ from males. Data presented as mean ± SD. Alan C. Burton's (1935; see manuscript reference list) weighted temperature equations are included on the figure.
